# Effectiveness of iodine-impregnated incise drapes for preventing surgical site infection in patients with clean or clean contaminated wounds: A systematic literature review and cost-consequence analysis

**DOI:** 10.1177/17504589221139603

**Published:** 2023-01-27

**Authors:** Katie Sworn, Edith Poku, Praveen Thokala, Anthea Sutton, Steve Foster, Ian Siddall, Henning Reuter

**Affiliations:** 1 School of Health and Related Research (ScHARR), University of Sheffield, Sheffield, UK; 2 3M UK PLC, Loughborough, UK; 3 Medical Solutions Division, 3M Deutschland GmbH, Neuss, Germany

**Keywords:** Iodine-impregnated incise drapes, Surgical site infection, Systematic review, Cost-consequence analysis, Infection control

## Abstract

**Background:**

Surgical site infection is a serious complication associated with significant morbidity, mortality and health care expenditure.

**Aims:**

To determine the clinical effectiveness and economic impact of using iodine-impregnated incise drapes for preventing surgical site infection.

**Methods:**

MEDLINE, Embase, Cochrane Library and CINAHL databases were systematically searched. Critical appraisal and synthesis of clinical evidence informed a decision analytical cost-consequence model.

**Findings:**

Nine studies were included in the systematic literature review. Evidence from cardiac surgery patients was considered appropriate to inform the cost analysis. The economic model evaluation estimated cost savings of £549 per patient with the iodophor-impregnated drape in the deterministic analysis and a mean cost saving per patient of £554,172 per 1000 in the probabilistic analysis.

**Conclusion:**

Using iodine-impregnated drapes in cardiac surgery patients may effectively reduce infections and provide cost-savings, but further research is required.

## Introduction

A Surgical Site Infection (SSI) occurs in the wound after a surgical procedure (Allegranzi et al 2016). Multiple risk factors may contribute to the development of SSIs. These include patient-related factors (eg: microbial colonisation); procedure-related factors (eg: use of antibiotics, surgical site preparation) and miscellaneous factors (eg: type of surgery, surgical room contamination) (Allegranzi et al 2016, Berríos-Torres et al 2017). SSIs are associated with significant morbidity, mortality and health care expenditure (Allegranzi et al 2016, Berríos-Torres et al 2017). The risks of SSIs are complex and often multifactorial, but are generally related to the patient’s health and type of surgery with a higher risk associated with surgical wounds that are contaminated, dirty or infected (Kamel et al 2011, see Table 1).

**Table 1:** Summary of classification of clean, clean-contaminated, contaminated and dirty or infected surgical wounds (Kamel et al 2011)

Classification of surgical wound	Clean	Clean-contaminated	Contaminated	Dirty or infected
Entry into respiratory, alimentary, genital, or urinary tracts	No	Yes, but under controlled conditions	Yes, but not under controlled conditions^a^	Yes, but not under controlled conditions^a^
Presence of contamination	No	Yes, eg: due to a breach in sterile operative technique or a major spillage of contents from any relevant tract	Yes, eg: due to the presence of devitalised tissue or contaminated contents from perforated tract	Yes, eg: due to the presence of devitalised tissue or contaminated contents from perforated tract
Presence of evident infection	No	No	Somewhat, inflammation without pus	Yes, inflammation with pus is evident

a Due to breach in the sterile operative technique or a major spillage of contents from any relevant tract.

Source: Kamel et al 2011.

Guidelines and standards of practice exist to reduce the risk of SSIs (NICE 2020, PHE 2019, WHO 2018). Recommended infection-reducing interventions can be implemented at preoperative, intraoperative or postoperative stages. A drape is a cover over the patient’s body. Surgical drapes provide a sterile operating field, reducing infection. An incise drape is an adhesive sheet which is used to isolate the operative site to reduce contamination of the surgical wound by theoretically acting as an antimicrobial barrier to the patient’s skin (Hesselvig et al 2020, Rezapoor et al 2018). The use of incise drapes is intraoperative and may or may not contain an antiseptic, such as iodophor (Kamel et al 2011).

Current NICE guidelines do not recommend the routine use of non-iodophor-impregnated incise drapes during surgery. If an incise drape is required, the guidelines recommend the use of an iodophor-impregnated drape unless the patient has an iodine allergy (NICE 2020: p9). Allegranzi et al (2016) determined that plastic adhesive incise drapes, with or without antimicrobial properties, should not be used (conditional recommendation) based on low and very low quality evidence. Berríos-Torres et al (2017) determined with weak certainty no benefit compared to no drape from limited but high-quality evidence. The evidence on the use of iodophor-impregnated drapes has been debatable (Anderson et al 2014, Berríos-Torres et al 2017, Nicholson et al 2020, Webster & Alghamdi 2015). A review by Webster and Alghamdi (2015) concluded that iodophor-impregnated drapes had no effect on SSI rates. Nicholson et al (2020) found iodine-impregnated drapes are beneficial in reducing postoperative SSI for all surgeries including contaminated surgeries. There is currently uncertainty about the costs and benefits, and understanding economic impact is vital to inform future tailored recommendations for infection-reducing interventions, for surgical intervention types and clean or clean-contaminated surgery.

A systematic literature review was undertaken to determine the effectiveness of iodine-impregnated drapes for the reduction of SSI risk following ‘clean or clean contaminated’ surgery, allowing an economic analysis to determine the cost impact of using iodophor-impregnated drapes in the UK in surgical specialities.

## Methods

**Figure 1:**
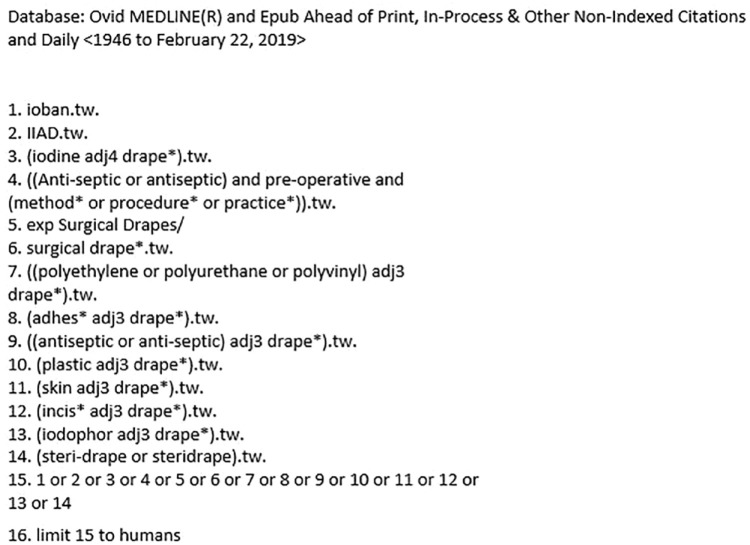
Example search MEDLINE search strategy

We describe the methods relating to the review of clinical effectiveness and cost-effectiveness analysis separately. We adhered to the synthesis without meta-analysis (SWiM) guidelines (Campbell et al 2020), also incorporating Preferred Reporting Items for Systematic Reviews and Meta Analysis (PRISMA) (Moher et al 2009) and the core principles for a systematic review of health interventions (Centre for Reviews and Dissemination 2009). Search terms from a Cochrane review (Webster & Alghamdi 2015) were used to develop a search strategy. Terms related to the intervention were ‘iodine impregnated self-adhesive plastic drapes (IIAD),’ ‘iodine-impregnated drape,’ ‘Ioban’ and ‘adhesive or surgical drapes’ (Figure 1). A preceding scoping search retrieved limited records when terms for surgery type, for example, surgical repair or abdominal surgery, were included. Therefore, searches were conducted in MEDLINE, Embase, the Cochrane Library and CINAHL up to 28 February 2019, with terms for the intervention and without date limits. We also examined reference lists of included studies.

Study selection for the review was based on pre-specified eligibility criteria as outlined in Table 2 that specified Population, Intervention, Comparator, Outcome, Study design and Language elements. One reviewer screened titles and abstracts of all records and selected potentially relevant studies. A random selection of titles and abstracts was double-checked for inclusion by a second reviewer. Subsequently, two reviewers independently completed study selection of full-text articles and differences were resolved by discussion within the team. Authors selected studies for economic analysis based on publication within the last ten years, providing the most recent and relevant clinical effectiveness evidence base.

**Table 2:** Inclusion and exclusion criteria

	Inclusion criteria	Exclusion criteria
Population	Patients, undergoing outpatient or inpatient surgery with a *clean or clean contaminated* wound^a^	Patients, undergoing outpatient or inpatient surgery with a *Contaminated or dirty or infected* wound^a^ Immunocompromised patients
Intervention	Antimicrobial incise drape, specified as 3M^™^ Ioban^™^ antimicrobial incise drape or iodine-impregnated drape - Used alone or in combination with other drapes	Antimicrobial incise drape, specified as 3M^™^ Ioban^™^ antimicrobial incise drape or iodine-impregnated drape - Not used as an incision drape
Comparator	Standard (ie: not iodine-impregnated) incision drape (polyethylene/polyurethane/polyvinyl) No drape	Non-adhesive drapes
Outcomes	Surgical site infection Adverse events (eg: skin reactions, sepsis, shock) Length of hospital stay Hospital re-admissions Health-related quality of life	Bacterial count Wound colonisation
Study Design	Systematic review Randomised controlled trial Non-randomised controlled trial	Case series
Language	English	Non-English

a Classifications of surgical wound types are summarised in Table 1.

A data extraction form was developed, piloted and subsequently finalised. Abstracted information included study and population characteristics (eg: study design, type of surgery and drape used, perioperative and intraoperative procedures, sample size, age, comorbidities) and reported outcomes of interest (eg: SSIs, adverse events). Data extraction was completed by one reviewer, then cross-checked for accuracy by a second reviewer. The Cochrane Risk of Bias tool (RoB 2) (Higgins et al 2019) and the Risk Of Bias In Non-randomised Studies of Interventions tool (ROBINS-I) (Sterne et al 2016) guided quality appraisal. To assess strength of clinical evidence, we applied a tool for narrative summary of results (Murad et al 2017) rather than the Grading of Recommendation, Assessment, Development and Evaluation (GRADE) tool (Schünemann et al 2013) as it was not appropriate to pool data.

Findings were presented narratively and in evidence tables. Meta-analysis was considered inappropriate due to heterogeneity in included studies. The narrative synthesis grouped results according to comparators: a standard adhesive incision drape (polyethylene/polyurethane/polyvinyl) or no drape or iodine-impregnated drape. There were no minimum criteria for studies for inclusion in a narrative synthesis. Heterogeneity was observed in tabulation of outcome effect estimates. Where data permitted, the risk ratios (RRs) with 95% confidence intervals (CIs) were calculated in Review Manager (RevMan version 5.3).

### Methods for estimating economic impact

**Figure 2:**
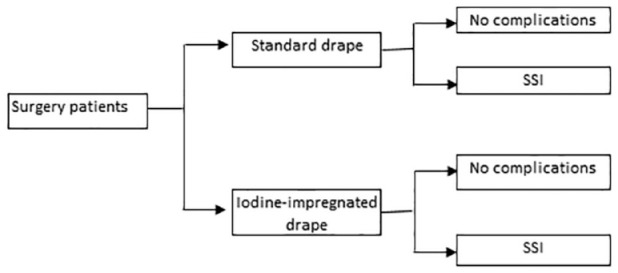
Model structure Abbreviation: SSI: Surgical Site Infection

A decision analytical cost-consequence model was developed using Microsoft Excel software (Microsoft Corporation) to estimate the economic impact of using iodine-impregnated drapes compared to standard drapes for surgical patients from systematic literature review sources. The economic analysis used the England and Wales National Health Service (NHS) perspective in the model. The structure of the model is shown in Figure 2.

The model assigned each surgical patient with a risk of SSI based on whether they had standard drape or iodine-impregnated drape and compared estimated costs for these groups. The systematic literature review identified the risk of SSIs (methods for review outlined in section Methods for estimating clinical effectiveness).

Costs were accrued through costs of drape (ie: iodine-impregnated or standard drape) and hospital treatment costs depended on whether the patients had SSI. Total costs were estimated as mean values of 10,000 probabilistic sensitivity analysis (PSA) runs, each run with a different estimate for the risks, and costs sampled from probability distributions representing uncertainty in the parameter estimates.

### Clinical effectiveness review

**Figure 3:**
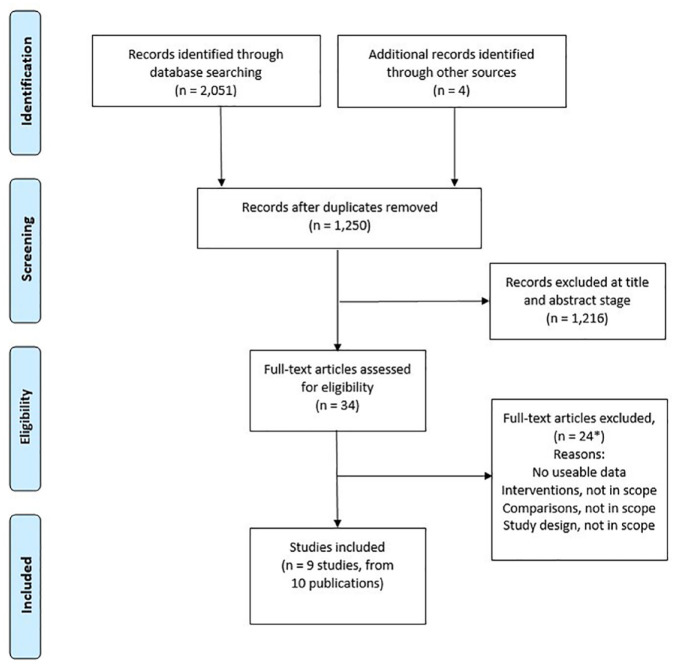
PRISMA flow diagram of study selection

A total of 1250 unique records were identified. Based on pre-specified selection criteria, nine primary studies related to ten publications, two randomised controlled trials (RCTs) and seven non-RCTs; n = 4370 participants, and one meta-review were eligible for inclusion (Figure 3). Included studies were heterogeneous in terms of study designs, definitions and assessments of SSI and did not allow for undertaking a meta-analysis. The eligibility criteria were outlined in a protocol. The only change to the protocol was to undertake a critical appraisal of included studies.

### Study characteristics

Included studies were diverse in study design and reported outcomes (Table 3). Two studies compared 3M^™^ Ioban^™^ antimicrobial incise drape with a standard or non-antimicrobial incision drape (Bejko et al 2015, Haliasos et al 2012). The remaining studies used a ‘no drape’ comparator (Dewan et al 1987, Karapinar & Kocatürk 2019, Moores et al 2017, Ritter & Campbell 1988, Segal & Anderson 2002, Swenson et al 2008, Yoshimura et al 2003). One RCT (Segal & Anderson 2002) did not specify whether the iodine-impregnated drape used was a 3M^™^ Ioban^™^ antimicrobial incise drape – included due to similarity of the properties of the drape and limited data identified. Two studies (Dewan et al 1987, Segal & Anderson 2002) were reported in a meta-review (Liu et al 2018) identified during database searching. Therefore, the meta-review did not provide additional data.

**Table 3:** Characteristics, risk of bias and outcomes of primary studies

Author, Publication year	Comparison	Study type and methods	Outcome definition and assessment	Country/setting	Type of surgery	Risk of bias, overall assessment	Sample size [mean age, years] Interventions	Reported outcomes	Findings: RR 95% CI/relevant data	
Bejko et al (2015)	3M™ Ioban™ antimicrobial incise drape versus standard or non-antimicrobial incision drape	Non-RCT Retrospective secondary analysis of database case–control Propensity matched score technique applied to examine incidence of SSI Cost analysis	A wound complicated by the presence of fever, raised white blood cells, positive bacterial cultures and need of antibiotic therapy, and/or in presence of local signs such as wound drainage, redness, skin discharge or dehiscence, and fat necrosis SSI was sub-divided into superficial SSI, when infection was limited to the subcutaneous tissue and deep SSI when the muscular fascia, bone and/or mediastinum is singularly or concurrently affected (authors reported that assessment of infection was in line with the Centres for Disease Control and Prevention classifications) Assessment: Retrospective study Follow-up, duration of hospitalisation and 90 days after discharge	Italy	Cardiac surgery	**Moderate**	1616/5100 participants were included in the study To ensure baseline comparability, a matched cohort of 1616 participants were identified based on propensity scoring for age, gender, diabetes mellitus, congestive heart failure, type of cardiac procedure (according to the cardiac pathology), chronic obstructive pulmonary disease, renal insufficiency (serum creatinine level of 1.5 mg/dl or greater), obesity, re-intervention and urgency, cross clamp, extracorporeal (EC) and total operative time. Ioban (n = 808 [*66.5* *±* *11.7* years]); Non-iodine-impregnated plastic cellulose adhesive drape (Hartmann International FolioDrape Cardiovascular set) (n = 808 [*66.2* *±* *11.5* years])	SSI (overall)	RR 0.28	0.16 to 0.50
								SSI (superficial)	RR 0.32	0.17 to 0.59
								SSI (deep)	RR 0.27	0.08 to 0.97
								VAC therapy	RR 0.26	0.12 to 0.53
								Costs	€773.495 cost saving in favour of Ioban	
Haliasos et al (2012)	3M™ Ioban™ antimicrobial incise drape versus standard or non-antimicrobial incision drape	Non-RCT Prospective study (no control). Examined incidence of SSI	Clear definition, not reported Authors examined cultures from drapes Assessment: Prospective study Follow-up period, 8 to 10 years.	England	Ventriculoperitoneal shunt insertions	**Serious**	75 *[age, not reported]* Ioban (n = 20); Non-iodine-impregnated drape (n = 55).	SSI	RR 0.53	0.03 to 10.66
Segal and Anderson (2002) Follow-up: 6 weeks post-operatively at scheduled timepoints (not specified)	One-step iodophor/alcohol water-insoluble film with iodine-impregnated drape iodophor/alcohol water-insoluble film (3M^™^ antimicrobial incise drape not specified; however, we made assumption properties were relevant to the intervention of interest	RCT Randomised trial of 4 patient groups Comparison of 2 case groups in our analysis) Chi-squared comparison analysis	A sternal surgical site showing signs of inflammation and positive bacterial cultures (authors reported that the assessment of infection was in line with the Centres for Disease Control and Prevention classifications) Assessment: Prospective study Data collected by nurse specialist or cardiovascular outcomes manager Follow-up, 6 weeks postoperatively	USA	High-risk open-heart surgery (non-emergency)	**Some concerns**	Of the 209 participants, 101 were grouped in the insoluble iodine prep group (intervention of interest [*60.9* years]). Reported age across the four intervention groups with no further information One-step iodophor/alcohol water-insoluble film with iodine-impregnated drape (n = 51); (group 4) iodophor/alcohol water-insoluble film (group 3) (n = 50). (Other non-relevant groups applied iodine paint and or scrub)	SSI	RR 2.94	0.32 to 27.33
Dewan et al (1987)	3M^™^ Ioban^™^ antimicrobial incise drape versus no drape	RCT Prospective case–control randomised clinical trial	A discharge of fluid or pus associated with a positive bacterial culture from the surgical wound or The presence of erythema of a third or 8 cm of the length of the wound and > I cm lateral to the wound margin Assessment: Prospective study Wound was assessed by an Infection Control Nurse; 3 to 4 days; 8 to 10 days and 3 weeks, post-surgery Bacterial cultures to confirm infection Expert advice from the surgeon to review decisions which were unclear [abdominal operation, including inguinal hernia repair]	New Zealand	Abdominal operation, including inguinal hernia	**High**	1016 [*age, not reported*] The study included participants aged 10 years and more. Ioban (n = 529) No drape (n = 487)	SSI (all wounds)	RR 0.97	0.62 to 1.53
								SSI (clean wounds)	RR 1.16	0.68 to 1.98
								SSI (clean contaminated wounds)	RR 0.92	0.58 to 1.46
Moores et al (2017) Follow-up 30 day post-operatively	3M^™^ Ioban^™^ antimicrobial incise drape versus no drape	Non-RCT Case-control study Prospective case-control analysis of SSI incidence	A wound infection occurring within 30 days of operation, affecting superficial, deep, or organ spaces or any purulent drainage, or culture-positive wound, or local inflammation that resulted in the	USA	Open ventral hernia repair with a retromuscular repair using synthetic mesh	**Serious**	104 Ioban (n = 48) [*61* *years*] No drape (n = 56) [*62* *years*]	SSI Outcomes were presented as surgical site occurrences, a broad category of	RR 5.82	0.29 to 118.27
			surgical re-opening of the wound (authors reported that assessment of infection was in line with the Centres for Disease Control and Prevention classifications) Prospective study Follow-up, 30 days [open ventral hernia repair]					infective and non-infective inflammatory conditions Of the seven surgical occurrences in the Ioban group, two were presented as superficial surgical site infections		
								Readmission	RR 1.17	0.31 to 4.42
								Adverse event (allergic reaction to Ioban)	RR 0.39	0.06 to 1.84
Swenson et al (2008)	3M^™^ Ioban^™^ antimicrobial incise drape versus no drape	Non-RCT Prospective secondary analysis study comparing case and control. Univariate and logistic regression analysis of factors associated with infection	All mesh infections in the first 30-day postoperative period, as well as surgical site infection (undefined) not related to the mesh A mesh infection was defined as infection that required operative removal of the mesh Assessment: Retrospective review of hospital databases (University of Virginia Health System American College of Surgeons National Surgical Quality Improvement Programme [ACS-NSQIP] 2002 to 2006) by surgical clinical nurses Follow-up, 30 days postoperatively	USA	Ventral or incisional hernia repair (including laparoscopic procedures, incarcerated hernias and recurrent hernia repair)	**Serious**	506 Ioban (n = 206) [*52.1* *±* *13.1* *years*]; No drape (n = 300) [*53.5* *±* *14.1* *years*]	SSI	RR 0.81	0.51 to 1.28
								Mesh infection	RR 0.90	0.49 to 1.63
Yoshimura et al (2003)	3M^™^ Ioban^™^ antimicrobial incise drape versus no drape	Non-RCT Retrospective evaluation of patients in secondary analysis Logistic regression analysis of risk factors for wound infection	The presence of local inflammation (ie: pain, swelling, redness or warmth) and a purulent drainage from the superficial incision with or without laboratory confirmation. Wound infections associated with intra-abdominal infections were not included in the analysis. Assessment: Retrospective study Follow-up, 30 days postoperatively	Japan	Liver resection for hepatocellular carcinoma	**Moderate**	296 Ioban (n = 122) [*61.1* *±* *8.4* *years*]; No drape (n = 174) [*63.1* *±* *7.5* *years*]	SSI	RR 0.27	0.10 to 0.77
Karapinar and Kocatürk (2019)	3M^™^ Ioban^™^ antimicrobial incise drape versus no drape	Non-RCT Retrospective case-control comparison Cost analysis	*‘Surgical site infection was defined as a condition that affects the skin, subcutaneous tissues, and other tissues above the fascia and is characterised by clinical evidence of an infection, purulent discharge, growth in wound culture, or the presence of inflammation findings from 3* *days to 30* *days after surgery’* p.2 Assessment of the presence of accompanying leukocytosis, elevated C-reactive protein levels and fever, antibiotics was initiated after consultation with an infectious disease specialist without waiting for the culture results. Follow-up, 1 month	Turkey	Anatomic pulmonary resection via thoracotomy (lobectomy, bilobectomy and pneumonectomy) without sterile wound draping was performed between 2013 and 2014 Whereas anatomic pulmonary resection was done with sterile wound drapes from 2015 to 2016	**Serious**	654 Ioban (n = 380) [58.24 ± 12.14] No drape (n = 274) [60.03 ± 11.72 *years*]	SSI	RR 0.32	0.16 to 0.63
								VAC	RR 0.22	0.06 to 0.78
Ritter and Campbell (1988)	3M^™^ Ioban^™^ antimicrobial incise drape only	Non-RCT Retrospective evaluation of cases (no control)	A postoperative draining wound and/ or positive bacterial culture from the joint (not skin) Assessment: Retrospective study Follow-up, at least 1 year	USA	Hip and knee arthroplasties	**Critical**	649 [*age, not reported*]		SSI 0.46% (n = 3/649)	

Abbreviations: CI, confidence interval; n, number; RCT, randomised controlled trial; RR, risk ratio; SSI, surgical site infection; VAC, vacuum-assisted therapy; CRP, C-reactive protein.

For Non-RCTs, the Risk Of Bias In Non-randomised Studies of Interventions (ROBINS-I tool) classifies a study as low (the study is comparable to a well-performed randomised trial with regard to a specific domain), moderate (the study is sound for a non-randomised study with regard to a domain but cannot be considered comparable to a well-performed randomised trial), serious (the study has some important problems) and critical risk of bias (the study is too problematic to provide any useful evidence on the effects of intervention).

For RCTs, domains covered by version 2 of the Cochrane risk of bias tool for randomised trials (RoB2) are bias due to confounding; bias in the selection of participants into the study; bias in the classification of interventions; bias due to deviations from intended intervention; bias due to missing data; bias in the measurement of outcomes and bias in the selection of the reported result. The RoB 2 tool classifies the overall risk of bias from low, some concerns to high.

Seven non-RCT studies were included in the review. With the exception of one prospective study (Haliasos et al 2012), the remaining non-RCTs were retrospective and included: a case–control study (Moores et al 2017); propensity score-matched analysis (Bejko et al 2015); secondary analysis of database records; analysis of risk factors for infection (Yoshimura et al 2003) and two retrospective evaluations of surgical practice (Karapinar & Kocatürk 2019, Ritter & Campbell 1988). Included studies were conducted in the USA (Moores et al 2017, Ritter & Campbell 1988, Segal & Anderson 2002, Swenson et al 2008); in England (Haliasos et al 2012), Italy (Bejko et al 2015), in Japan (Yoshimura et al 2003), in Turkey and in New Zealand (Dewan et al 1987). Publication dates were between 1987 and 2019. Swenson et al (2008) included both in-patients and outpatients, while remaining studies focused on hospital in-patient settings. In addition, patients received different preoperative or perioperative management, for example, antibiotics were administered in six studies (Bejko et al 2015, Moores et al 2017, Ritter & Campbell 1988, Segal & Anderson 2002, Swenson et al 2008, Yoshimura et al 2003).

### Risk of bias assessments

An assessment of the risk of bias and certainty of the evidence was undertaken. Two non-RCTs comparing iodine-impregnated drape versus standard drape (Bejko et al 2015, Haliasos et al 2012) were rated as moderate and serious risk of bias, respectively. The one study (Bejko et al 2015) adjusted for confounders and selection of participants through propensity-matched scoring. Haliasos et al (2012) presented limited information relating to how confounding and bias in outcome measurements were addressed.

Among studies with a ‘no drape’ comparator, one RCT (Segal & Anderson 2002) and one non-RCT (Yoshimura et al 2003) had an overall assessment of moderate and some concerns, respectively. Three non-RCTs received a serious risk of bias rating (Karapinar & Kocatürk 2019, Moores et al 2017, Swenson et al 2008), while another RCT (Dewan et al 1987) had a high risk of bias assessment. Dewan et al (1987) performed poorly in the selection of participants post-randomisation and outcome reporting.

Main concerns across non-RCTs were related to several areas. These included, addressing likely confounders (Swenson et al 2008, Yoshimura et al 2003), the definition of intervention groups (Ritter & Campbell 1988) and the potential for bias in the selection of retrospective secondary analysis methods (Karapinar & Kocatürk 2019, Swenson et al 2008, Yoshimura et al 2003). Full details of the assessment are available on request.

### Surgical site infection outcomes

Included studies were grouped by comparators: standard drape (ie: not iodine-impregnated) or no drape (Table 3) because it would not be clinically appropriate to combine these groups.

### Comparison: iodine-impregnated drape versus standard incision drape

Two non-RCTs (Bejko et al 2015, Haliasos et al 2012) and one RCT (Segal & Anderson 2002) contributed data for this comparison. In the study evaluating patients with high-risk open-heart surgery (n = 101) by (Segal & Anderson 2002), SSI outcomes did not favour the intervention compared to one-step iodophor/alcohol water-insoluble film (RR 2.94 95% CI 0.32 to 27.33, p value, not reported). However, in patients who underwent cardiac surgery (n = 1616 patients), the use of the intervention showed a reduction of 68% to 72% in SSIs (superficial: RR 0.31, 95% CI 0.16 to 0.58; deep: RR 0.27, 95% CI 0.08 to 0.97; overall: RR 0.27, 95% CI 0.15 to 0.48, p = 0.001) (Bejko et al 2015). Patients receiving ventriculoperitoneal shunt insertions showed non-significant benefits (RR 0.53, 95% CI 0.03 to 10.66) in favour of the intervention (n = 20 patients) compared to standard drapes (n = 55 patients) (Haliasos et al 2012).

### Comparison: iodine-impregnated drape versus no drape

One RCT (Dewan et al 1987) and five non-RCTs (Karapinar & Kocatürk 2019, Moores et al 2017, Ritter & Campbell 1988, Swenson et al 2008, Yoshimura et al 2003) provided data. The study by Ritter & Campbell 1988 did not specify a comparator.

Three studies in patients receiving liver resection (n = 296; SSI: RR 0.27, 95% CI 0.10 to 0.77, p = 0.0096) (Yoshimura et al 2003); pulmonary resection (n = 654; SSI: RR 0.32, 95% CI 0.16 to 0.63, p = 001) (Karapinar and Kocatürk 2019) and hip and knee arthroplasties (n = 3/649; SSI 0.46%) (Ritter & Campbell 1988) reported statistically significant results in favour of the intervention (Table 3). The remaining studies presented unclear or non-significant results about the benefit conferred by intervention in patients receiving abdominal operations including hernia repair (RCT: n = 1016; clean SSIs RR 1.16, 95% CI 0.68 to 1.98, clean contaminated SSIs RR 0.92, 95% CI 0.58 to 1.46; all SSIs RR 0.97, 95% CI 0.62 to 1.53) (Dewan et al 1987) and non-RCTs: n = 104; RR 5.82, 95% CI 0.29 to 118.27, p = 0.25 (Moores et al 2017) and n = 506: SSI RR 0.81, 95% CI 0.51 to 1.28, p = 0.36) (Swenson et al 2008).

### Other outcomes

Compared to standard drapes, the use of iodine-impregnated drapes (Ioban) reduced vacuum-assisted therapy in cardiac surgery patients (RR 0.22, 95% CI 0.06 to 0.78) (Bejko et al 2015). Swenson et al (2008) found that mesh infections were lower in the Ioban group compared with no drape group (RR 0.90, 95% CI 0.49 to 1.63). Moores et al (2017) compared Ioban to no drape reported readmission rates (RR 1.17, 95% CI 0.31 to 4.42) and adverse events (RR 0.39, 95% CI 0.06 to 1.84).

### Certainty of findings

Based on GRADE recommendations (Murad et al 2017), the existing heterogeneity of results meant it was not appropriate to pool effect estimates. Overall, the certainty of available evidence was rated as very low. Full details of the assessment are available on request.

### Selection of studies for the economic evaluation

Only studies with a low risk of bias should be considered to inform the economic evaluation. Based on the assessment of study quality (ROBINS-1, RoB 2 tools) and certainty of evidence (Murad et al 2017), the study by Bejko et al (2015) was considered the most appropriate evidence for the cost-consequence analysis. Significant heterogeneity and limited methodological quality were noted in the remaining studies included in the systematic literature review.

## Economic analysis

### Summary of model parameters

A summary of the model parameters is provided in Table 4 along with their sources.

**Table 4:** Summary of model parameters

Parameter	Mean	Distribution	Source
**SSI risks (per patient)**			
Standard drape	6.5%	Beta (53,755)	Bejko et al 2015
Iodine-impregnated drape	1.9%	Beta (15,793)	Bejko et al 2015
**Costs (in £)**			
Standard drape cost per patient	£18.72	–	3M
Iodine-impregnated drape cost per patient	£31.27	–	3M
SSI costs	£12,198	Gamma (20,060)	Jenks et al 2014, Curtis and Burns 2018

SSI: Surgical Site Infection.

## Risks of SSI

The study examined the efficacy of iodine-impregnated drape compared to standard drape (ie: not iodine-impregnated) in preventing SSI in cardiac surgery using propensity-matched analysis of 808 patients in each arm (Bejko et al 2015). Data suggested that the incidence of SSI was significantly higher in the standard drape group compared to the iodine-impregnated drape group (6.5% versus 1.9 %) (p = 0.001).

## Costs

### Costs of drapes

The cost of standard drape per patient was estimated as £18.72, assuming that the 3M surgical incise drape pack is similar to the Hartman drape used in the study by Bejko et al 2015. At the time of the study, the cost of using an iodine-impregnated drape per patient was provided by 3M as £31.27.

### Costs of SSI

The cost of SSI included costs of diagnosis, treatment and the costs associated with increased length of stay (Badia et al 2017). The data from Jenks et al (2014) were inflated using the Hospital and Community Health Services Inflation Index (HCHS) inflation indices to reflect current prices resulting in a median cost of SSI of £12,198 (95% CI £8,952, £16,180).

## Base case results

The deterministic analysis suggested the use of iodine-impregnated drape resulted in cost savings of £549 per patient compared to the use of a standard drape, which is mainly due to the reduced risk of SSI when using the iodine-impregnated drape. That is, although the cost of iodine-impregnated drape is higher than the standard drape, the substantial costs associated with SSI results in overall cost savings as there is a lower chance of an SSI in the iodine-impregnated drape than that in the standard drape.

**Figure 4:**
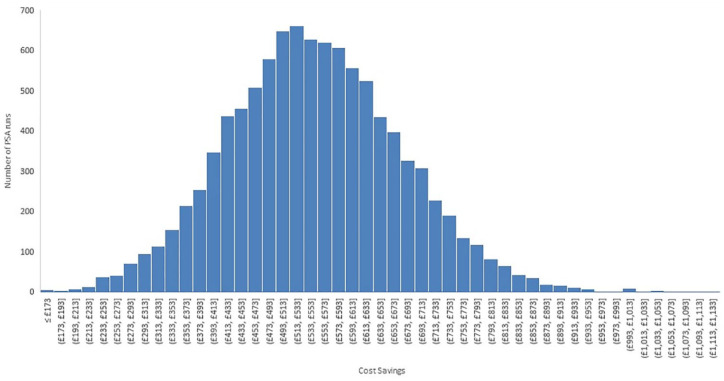
Histogram of cost savings from the 10,000 probabilistic sensitivity analysis runs

The results of the probabilistic sensitivity analysis (PSA) are similar to the deterministic analyses results with an average cost savings of £554 per patient. Also, the iodine-impregnated drape arm was cost saving in each of the 10,000 PSA runs. Figure 4 shows the histogram of the cost savings from the PSA.

## Scenario analyses

Scenario analyses were performed using alternative values of the parameters to understand their impact on cost savings. A scenario analysis was performed assuming that the costs of SSI are £20,000, which was selected as a realistic upper bound on the costs of SSI. Another scenario analysis used costs of SSI estimated in the NICE clinical guidelines (NG125) (NICE 2019), which reported lower costs of £7,515. Due to the limited availability of clinical effectiveness data and the selection of a single moderate-quality study to provide clinical effectiveness data, another scenario analysis was performed assuming lower SSI rate in the standard drape arm of 4.5% to estimate the impact of lower baseline risk of SSI on costs. In the scenario assuming higher SSI costs of £20,000, the mean cost savings increased to £907 per patient. In the scenario assuming lower SSI costs of £7,515, the mean cost savings decrease to £333 per patient. In the scenario assuming lower SSI rate of 4.5% in the standard drape arm, the mean cost savings decrease to £305 per patient.

## Discussion

A systematic literature review was undertaken to identify the best available clinical effectiveness evidence to determine the cost impact of using iodine-impregnated drapes for the prevention or reduction of SSIs. Nine studies were included in the review. Extensive heterogeneity in study design, populations and quality were observed, for example, length of surgery, a key factor associated with the risk of infection, with cardiac surgeries (coronary artery bypass graft (CABG) and cardiac surgery) being the longest with an average of five hours (PHE 2018). Considering the mixed quality of studies, settings, surgery types, study designs and dates, it was not possible to determine the overall effectiveness of SSI outcomes across all surgical intervention types. However, the economic analysis helps to identify potential cost savings for cardiac surgery based on the assessment of quality and certainty of available clinical evidence.

Currently, there is uncertainty relating to the intra-operative use of iodophor-impregnated drapes in preventing SSIs (Allegranzi et al 2016, Anderson et al 2014, Berríos-Torres et al 2017, Webster & Alghamdi 2015). A previous review reported the intervention had no effect on the SSI rate compared to no drape (RR 1.03, 95% CI 0.06 to 1.66, p = 0.89) (WHO 2018). A review by Nicholson et al (2020) found iodine-impregnated drapes are beneficial in reducing postoperative SSI for all surgeries including clean-contaminated and contaminated surgeries. Recent UK guidelines recommend the use of an iodophor-impregnated drape if a plastic adhesive drape is required unless there are any contraindications such as iodine allergy (NICE 2020: p9). A single study (Bejko et al 2015) was considered high enough quality and power to detect differences between comparator groups. Propensity score weighting addressed potential confounders. Cardiac surgery patients represent a large surgical population at high risk of SSI, associated with very significant adverse postoperative clinical outcomes and treatment costs. Therefore, the cost impact of iodophor-impregnated drapes was modelled using data from Bejko et al.

## Limitations

Included studies were heterogeneous, and meta-analysis of pooled results was deemed inappropriate. Results should be interpreted with caution due to variability in baseline standards (such as the use of antibiotics or preoperative wound cleansing) which would affect postoperative outcomes. The review included older papers pre-dating newer standards for study design and reporting – negatively affecting quality appraisal ratings. An adapted GRADE approach (Murad et al 2017) indicated the evidence was of very low certainty overall. The impact of missing recent publications is unclear. However, searches using the terms ‘iodine-impregnated incise drape,’ ‘ioban’ and ‘surgical site infection’ in PubMed on 22 July 2021 found no additional relevant publications. The Nicholson et al (2020) review was identified subsequently but did not identify new studies meeting our inclusion criteria.

## Conclusion

The clinical review consisted of a robust literature search and critical appraisal. The heterogeneous evidence base contained few recent, large-scale studies of high quality. Our economic analyses were based on a non-randomised study (Bejko et al 2015). Ioban resulted in an overall saving of £554,172 per 1000 adult patients, that is, an average cost saving of £554 per patient compared to standard drape. The results were robust to sensitivity analyses performed on the baseline SSI risks and unit cost of SSI.

There are around 30,000 surgery patients treated in England and Wales annually who could potentially benefit from iodine-impregnated drapes. Based on the cost model developed, substituting standard drapes with iodine-impregnated drapes would result in an estimated cost savings of around £17 million per annum. However, this analysis is based on a single study. Future large multi-centre SSI prevention trials are needed to explore the use of incising drapes. Future studies could verify the benefits and cost-savings of antimicrobial incise drapes for different types of cardiac surgery.

## Declarations

**Ethical approval:** No ethical approval was required because the study included publicly available information
